# Maternal and perinatal outcomes in adolescents and women with advanced maternal age

**DOI:** 10.11606/s1518-8787.2025059006538

**Published:** 2025-10-20

**Authors:** Silvana Granado Nogueira da Gama, Arthur Orlando Corrêa Schilithz, Maria do Carmo Leal, Talita Teresa do Carmo, Mariza Miranda Theme-Filha, Denise Cavalcante de Barros, Glauce Cristine Ferreira Soares, Katrini Guidolini Martinelli

**Affiliations:** I Fundação Oswaldo Cruz. Escola Nacional de Saúde Pública. Rio de Janeiro, RJ, Brasil; II Instituto Nacional de Propriedade Industrial. Rio de Janeiro, RJ, Brasil; III Universidade de São Paulo. Escola de Artes, Ciências e Humanidades. São Paulo, SP, Brasil; IV Universidade Federal do Sul da Bahia. Centro de Formação em Ciências da Saúde. Teixeira de Freitas, BA, Brasil

**Keywords:** Maternal age, Pregnancy in adolescence, Pregnancy Complications, Pregnancy Outcome, Infant, Premature

## Abstract

**OBJECTIVE:**

To analyze the association between age extremes and maternal and perinatal outcomes in births in the state of Rio de Janeiro, Brazil.

**METHODS:**

Data from the Birth in Brazil II: National Survey on Abortion, Delivery, and Birth (2021–2023) were used. A total of 1,734 postpartum women were included, with live or stillborn newborns, interviewed in the immediate postpartum period. Information was obtained from interviews, clinical records, and prenatal cards. Multiple logistic regression was carried out to evaluate associations between maternal age and outcomes, having women aged 20–34 years as reference.

**RESULTS:**

Delivery in adolescents represented 10.1% of the participants, while women with advanced maternal age (AMA ≥ 35 years) corresponded to 18.0%. Adolescents were less likely to receive guidance as to reference maternity hospital (odds ratio — OR = 0.68) and to have a companion at all times (OR = 0.77), in addition to presenting lower prevalence of gestational diabetes (OR = 0.26). In turn, women with advanced maternal age were more often guided on reference maternity hospitals (OR = 1.46) and presented a higher risk of gestational hypertension (OR = 1.78), gestational diabetes (OR = 1.87), placental abruption (OR = 3.30), and severe maternal morbidity (OR = 1.84). In perinatal outcomes, adolescents had a higher risk of perinatal death (OR = 4.52) and spontaneous early-term delivery (OR = 1.47); however, there was a lower probability of early-term delivery by obstetric intervention (OR = 0.48). Newborns of women with advanced maternal age presented higher risk of 5-minute Apgar index < 7 (OR = 3.05) and lower chance of congenital syphilis (OR = 0.34).

**CONCLUSIONS:**

Adolescents were provided worse care compared to adults, while women with advanced maternal age presented a higher frequency of age-related complications. These results highlight the need for special care, particularly considering the increase in deliveries in women with advanced age.

## INTRODUCTION

The extreme age groups of reproductive life have been widely studied due to the increased risk of obstetric and perinatal complications. In Brazil, in 2022, about 30% of live births (LB) were from women in extreme age groups: 18.0% had advanced maternal age (AMA ≥ 35 years) and 12.3% were adolescents (< 20 years), according to data from the Live Birth Information System (*Sistema de Informações sobre Nascidos Vivos* – SINASC)^1^.

Maternity postponement has become a common choice among women in recent decades, driven by the pursuit of financial stability and career advancement^2^. However, pregnancy in women with AMA is associated with a higher risk of adverse outcomes — such as gestational hypertension, antepartum hemorrhage, gestational and pregestational diabetes as well as placental abnormalities^3,4^. Among perinatal outcomes, we highlight a higher incidence of stillbirth, preterm birth, macrosomia, and the need for hospitalization in a neonatal intensive care unit (NICU)^5,6^.

Despite the reduction in the proportion of teenage pregnancy, this group still demands special care due to the higher risk of perinatal death, early-term delivery, and fetal growth restriction, especially in younger adolescents^7–10^. In addition, among the negative maternal outcomes are anemia, hypertensive diseases of pregnancy, postpartum hemorrhage, and lack of social support^10,11^. Researchers also associate extreme maternal ages with a higher risk of severe maternal morbidity, including potentially fatal conditions such as near miss and maternal mortality^12^.

Furthermore, outcomes, such as syphilis, human immunodeficiency virus (HIV), severe maternal morbidity, congenital syphilis, preterm and early-term birth according to type of delivery, are still poorly investigated, especially in the Brazilian context. The policy on childbirth humanization, implemented by *Rede Cegonha* [Stork Network] (a program developed by the Federal Government aiming at labor care humanization), emphasizes soft technologies, such as linking pregnant woman to the maternity hospital, presence of companion, skin-to-skin contact, and early breastfeeding, to promote maternal and neonatal well-being^13^. In this study, we aimed to analyze the association between age extremes and maternal and perinatal outcomes in births in the state of Rio de Janeiro (RJ), Brazil, between 2021 and 2023.

## METHODS

This is an analysis of the study "Birth in Brazil II: National Survey on Abortion, Delivery, and Birth (*Nascer no Brasil II* – NBII)," which used specific data from the state of RJ corresponding to the period from 2021 to 2023.

The study population of NBII consists of women hospitalized due to delivery or abortion in the sampled hospitals. The sample was selected in two stages. The first stage corresponded to the hospitals and considered the number of annual LB, the Brazilian macroregion to which they belonged, and the type of hospital management — public, private, or mixed. The second stage of the sample concerned the postpartum women selected for the study. A total of 30 and 50 postpartum women were interviewed in hospitals with 100–499 and ≥ 500 LB/year, respectively. The NBII did not include those with communication difficulties due to severe mental disorder; those who did not understand Brazilian Portuguese; hearing-impaired women; those with triplet pregnancy or more; and those hospitalized for delivery by court order. Sample weights were calculated based on inclusion probabilities and calibrated by SINASC, 2022. The NBII study protocol is published in Leal et al.^14^.

For the present study, which focuses exclusively on the state of RJ, the postpartum women were distributed in 29 hospitals in 18 municipalities. The hospital interviews were conducted in the immediate postpartum period, between November 2021 and June 2023, with the following adaptations: widening the sample (50 to 90 postpartum women) in public and mixed hospitals with ≥ 500 LB/year and distinguishing the municipality of Rio de Janeiro (MRJ) and the other municipalities of the metropolitan region (MR) and small cities (SC). Calibration was carried out using the groups composed of the stratum combination (which combines the number of annual births of the facility, macroregion, type of hospital management, and the location of the hospital in the metropolitan region or small cities), type of delivery (vaginal, cesarean section), and women's age (< 20, 20–34, ≥ 35). For this analysis, in addition to the eligibility criteria already provided for NBII, the following were also excluded: women admitted for abortion, those who reported to be Indigenous and Asian, due to the small number of cases, and those who had twin pregnancies, due to their increased risk of adverse outcomes.

Information obtained from the interview with the postpartum women at hospital admission, the prenatal card, and the maternal and neonatal medical records were used. The outcomes analyzed were preferably collected from the prenatal card and maternal medical records; when not available, the interview with the pregnant woman was used.

There is a single exposure variable in this study: maternal age (< 20 — adolescents/20–34/≥ 35 years — advanced maternal age). The other independent variables are presented for the characterization of the study population, as follows: Skin color (white/Black or mixed-race); marital status (without a partner/with a partner); pregestational body mass index (BMI) (low weight/eutrophic/overweight/obesity); number of previous deliveries (0/1/≥ 2); intention of getting pregnant (intended to get pregnant/intentions varied or were unclear/had no intention of getting pregnant); prenatal care onset (first/second or third trimester/absence of prenatal care); adequacy of the number of prenatal care appointments for gestational age (no/yes); type of delivery (vaginal/cesarean section); financing of delivery (public/mixed/private); hospital location (metropolitan region/small cities).

Multiple outcomes were presented, separated between maternal and fetal. The dependent variables that represent maternal outcomes were: traveling to the maternity hospital (no/yes); guidance as to visiting the maternity hospital — link (no/yes); presence of companion (at all times/sometimes/had no companion at all); gestational hypertension — chronic hypertension, preeclampsia, eclampsia, or HELLP (hemolysis, elevated liver enzymes, low platelet count) syndrome (no/yes); placental abruption (no/yes); placenta accreta/increta/percreta (no/yes); maternal syphilis diagnosis (no/yes); HIV infection (no/yes); and severe maternal morbidity — potentially life-threatening conditions or maternal near miss (no/yes).

The fetal and neonatal outcomes included in the analysis were: perinatal death (no/yes); congenital syphilis (no/yes); intrauterine growth restriction (no/yes); low birth weight < 2,500 grams (no/yes); gestational age at birth (≤ 33 weeks/34–36 weeks/37–38 weeks/≥ 39 weeks); term at birth (≥ 39 weeks/spontaneous preterm/induced preterm or antepartum cesarian section/spontaneous early-term/induced early-term or antepartum cesarian section); 5-minute Apgar index < 7 (no/yes); skin-to-skin contact at the delivery room (no/yes); breastfeeding at the first hour (no/yes); the newborn was kept with the mother from birth to rooming-in or room (no/yes); and newborn's admission or transfer to NICU (no/yes).

The absolute and relative frequencies of maternal characteristics, maternal, fetal, and neonatal outcomes were presented. The differences in proportions between the groups (maternal age groups) were tested by Rao-Scott χ^2^ test and 95% confidence interval (95%CI).

Univariate and multivariate logistic regression models were developed to analyze the association between maternal age (exposure) and maternal, fetal, and neonatal outcomes through crude and adjusted odds ratio (OR). For potential confounding control, the adjustment variables were selected through theoretical plausibility. The adjustment of the multivariate model for maternal outcomes was composed of the variables: skin color, number of previous deliveries, and residence region. For fetal and neonatal outcomes, the adjustment was made for: skin color, number of previous deliveries, residence region, and financing of delivery.

The complex design of the sampling was taken into account throughout the statistical analysis. Each selection stratum went through a calibration process due to basic sample weights to ensure that the distribution of postpartum women was similar to that observed in the births of the population sampled in 2022, deriving weighted percentages.

## RESULTS

We included 1,734 postpartum women in the immediate postpartum period in the analyses, 10.1% of whom were adolescents and 18.0% were women with advanced maternal age. Most interviewees reported to be Black/mixed-race (69.6%), to have a partner (81.1%), be eutrophic (41.9%), without previous delivery (43.7%), having started prenatal care in the first trimester (78.6%), with the number of prenatal care appointments adequate for gestational age (67.3%), with public financing of delivery (63.3%), delivery in hospitals in the metropolitan region (76.1%), and cesarian section as type of delivery (58.5%) (Table 1).

**Table 1:** Sociodemographic and reproductive characteristics of postpartum women according to maternal age group. Rio de Janeiro, Brazil.

Characteristics		Age group (years)			Total (n = 1,732)	p-value^a^
		< 20 (n = 176)	20–34 (n = 1,228)	≥ 35 (n = 312)		
		%	%	%	%	
**Adequacy of prenatal care appointments (n = 1,656)**						
	no	76 (44.2)	401 (33.7)	65 (22.0)	542 (32.7)	0.002
	yes	96 (55.8)	788 (66.3)	230 (78.0)	1,114 (67.3)	
**Type of delivery (n = 1,733)**						
	vaginal	107 (58.8)	526 (42.5)	86 (27.6)	719 (41.5)	< 0.0001
	cesarean section	75 (41.2)	712 (57.5)	226 (72.4)	1,013 (58.5)	
**Financing of delivery (n = 1,734)**						
	public	145 (79.7)	812 (65.5)	140 (44.9)	1,097 (63.3)	< 0.0001
	mixed	30 (16.5)	154 (12.4)	34 (10.9)	218 (12.6)	
	private	7 (3.8)	273 (22.1)	138 (44.2)	418 (24.1)	
**Hospital location (n = 1,922)**						
	metropolitan region	138 (75.8)	935 (75.5)	246 (78.6)	1,319 (76.1)	0.561
	small cities	44 (24.2)	304 (24.5)	67 (21.4)	415 (23.9)	

BMI: body mass index (kg/m^2^).

a p-value of the Rao-Scott χ^2^ test.

When comparing age extremes, adolescents presented higher and significant percentages for Black/mixed-race skin color, having no partner, low weight, no intention of getting pregnant, without previous delivery, onset of prenatal care in the second/third trimester, public financing of delivery, and vaginal delivery. Conversely, women with AMA were mostly white, had a partner, were overweight or obese, had more intention of getting pregnant, two or more previous deliveries, started prenatal care in the first trimester, had greater adequacy as for the number of appointments, with private financing of delivery, and more cesarean sections.

In Table 2, we show gestational morbidities and maternal outcomes by age group. Traveling in search of labor care was identified in 12.1% of the interviewees, a value that almost doubles among adolescents, accounting for 23.6%. Only 17.7% of the postpartum women reported having been guided on visiting the maternity hospital for delivery, a worse result among adolescents, accounting for 12.6%. The presence of companion at all times of the woman's hospitalization was reported by 70.5% of the postpartum women, a similar value among the age groups. Gestational hypertension was identified in 16.1% of the interviewees, being lower in younger women (12.6%) and higher among the older (21.1%). Similarly, diabetes was more frequent in AMA (13.7%) as well as severe maternal morbidity (18.2%). The occurrence of placenta accreta/increta/percreta was low, identified in 0.5% of the women. The diagnosis of maternal syphilis was 14.3%, with a higher frequency in adolescents, 17.0%

**Table 2:** Gestational morbidities and maternal outcomes according to the postpartum women's age group. Rio de Janeiro, Brazil.

Maternal outcomes		Age group			Total (n = 1,732)	p-value^a^
		< 20 (n = 176)	20–34 (n = 1,228)	≥ 35 (n = 312)		
		%	%	%	%	
**Placental abruption (n = 1,732)**						
	no	176 (97.2)	1,230 (99.3)	306 (98.1)	1,712 (98.8)	0.080
	yes	5 (2.8)	9 (0.7)	6 (1.9)	20 (1.2)	
**Placenta accreta/increta/percreta (n = 1,734)**						
	no	182 (100.0)	1,236 (99.8)	307 (98.1)	1,725 (99.5)	0.003
	yes	0 (0.0)	3 (0.2)	6 (1.9)	9 (0.5)	
**Diagnosis of maternal syphilis (n = 1,733)**						
	no	151 (83.0)	1,036 (83.6)	299 (95.8)	1,486 (85.7)	< 0.001
	yes	31 (17.0)	203 (16.4)	13 (4.2)	247 (14.3)	
**HIV infection (n = 1,734)**						
	no	181 (99.5)	1,228 (99.1)	311 (99.4)	1,720 (99.2)	0.687
	yes	1 (0.5)	11 (0.9)	2 (0.6)	14 (0.8)	
**Severe maternal morbidity (n = 1,733)**						
	no	157 (86.7)	1,080 (87.2)	256 (81.8)	1,493 (86.2)	0.107
	yes	24 (13.3)	159 (12.8)	57 (18.2)	240 (13.8)	

Gestational hypertension: chronic hypertension, hypertensive disorders of pregnancy, preeclampsia, eclampsia, and HELLP.

Severe maternal morbidity: potentially life-threatening conditions or maternal near miss.

a p-value of the Rao-Scott χ^2^ test.

Regarding perinatal outcomes, we observed that 1.2% of births resulted in perinatal death, with this percentage being higher among adolescents (3.3%), although without statistical significance. Congenital syphilis was diagnosed in 7.9% of the cases, with a higher prevalence among the births of adolescents (9.4%). The proportion of preterm birth (< 37th gestational week) was 10.3%. Nevertheless, preterm birth before the 33rd week was more common among adolescents (6.1%), while in AMA, late preterm birth was more frequent (42.6%). Among adolescents, spontaneous preterm birth was more prevalent (7.2%), whereas preterm birth by induction or antepartum cesarean section (7.3%) was prevalent among AMA.

The prevalence of 5-minute Apgar index < 7 was similar among age groups. Skin-to-skin contact in the delivery room and breastfeeding in the first minute occurred in less than 60% of cases, without statistically differing between age groups. Regarding the permanence of newborns with mothers from birth to their room, 65.5% of the interviewees reported having stayed with their children, with a higher proportion observed among adolescents (73.6%). Likewise, hospitalization in NICU was less frequent among adolescents’ children and more prevalent among children of women aged 35 years or older, but without statistical significance (Table 3).

**Table 3:** Fetal and neonatal outcomes according to the postpartum women's age group. Rio de Janeiro, Brazil.

Fetal and neonatal outcomes		Age group			Total (n = 1,732)	p-value^a^
		< 20 (n = 182)	20–34 (n = 1,228)	≥ 35 (n = 312)		
		%	%	%	%	
**Perinatal death (n = 1,732)**						
	Yes	6 (3.3)	10 (0.8)	4 (1.3)	20 (1.2)	0.095
	No	176 (96.7)	1,228 (99.2)	308 (98.7)	1,712 (98.8)	
**Congenital syphilis (n = 1,732)**						
	yes	17 (9.4)	111 (9.0)	8 (2.6)	136 (7.9)	0.031
	no	164 (90.6)	1,127 (91.0)	305 (97.4)	1,596 (92.1)	
**Intrauterine growth restriction (n = 1,732)**						
	yes	4 (2.2)	12 (1.0)	4 (1.3)	20 (1.2)	0.285
	no	177 (97.8)	1,227 (99.0)	308 (98.7)	1,712 (98.8)	
**Low birth weight (< 2,500 grams) (n = 1,733)**						
	yes	21 (11.5)	113 (9.1)	28 (8.9)	162 (9.3)	0.596
	no	161 (88.5)	1,125 (90.9)	285 (91.1)	1,571 (90.7)	
**Gestational weeks (n = 1,734) (weeks)**						
	≤ 33	11 (6.1)	20 (1.6)	10 (3.2)	41 (2.4)	
	34–36	14 (7.7)	98 (7.9)	25 (8.0)	137 (7.9)	0.022
	37–38	47 (26.0)	375 (30.3)	133 (42.6)	555 (32.0)	
	≥ 39	109 (60.2)	746 (60.2)	144 (46.2)	999 (57.7)	
**Type of preterm birth (n = 1,732)**						
	≥39 weeks	109 (60.6)	746 (60.2)	144 (46.0)	999 (57.7)	
	Spontaneous preterm birth	13 (7.2)	56 (4.5)	12 (3.8)	81 (4.7)	
	Induced preterm birth or antepartum cesarean section	12 (6.7)	62 (5.0)	23 (7.3)	97 (5.6)	0.015
	Spontaneous early-term birth	38 (21.1)	168 (13.6)	45 (14.4)	251 (14.5)	
	Induced early-term birth or antepartum cesarean section	8 (4.4)	207 (16.7)	89 (28.4)	304 (17.6)	
**5-minute Apgar index (n = 1,722)** b						
	≥ 7	170 (96.6)	1,208 (97.7)	292 (94.2)	1,670 (97.0)	0.125
	< 7	6 (3.4)	28 (2.3)	18 (5.8)	52 (3.0)	
**Skin-to-skin contact in the delivery room (n = 1,718)** b						
	yes	105 (59.7)	716 (58.1)	176 (56.8)	997 (58.0)	0.752
	no	71 (40.3)	516 (41.9)	134 (43.2)	721 (42.0)	
**Breastfeeding in the first 24 h (n = 1,714)** b						
	yes	108 (61.0)	714 (58.1)	157 (51.0)	979 (57.1)	0.265
	no	69 (39.0)	515 (41.9)	151 (49.0)	735 (42.9)	
**Newborn was kept with the mother from birth to their room (n = 1,712)** b						
	yes	128 (73.6)	813 (66.3)	181 (58.0)	1,122 (65.5)	0.005
	no	46 (26.4)	413 (33.7)	131 (42.0)	590 (34.5)	
**ICU admission/newborn transfer (n = 1,724)** b						
	yes	13 (7.3)	99 (8.0)	38 (12.2)	150 (8.7)	0.286
	no	164 (92.7)	1,137 (92.0)	273 (87.8)	1,574 (91.3)	

Gestational hypertension: chronic hypertension, hypertensive disorders of pregnancy, preeclampsia, eclampsia, and HELLP.

Severe maternal morbidity: potentially life-threatening conditions or maternal near miss.

a p-value of the Rao-Scott χ^2^ test.

b Only for women with live births.

In Table 4, we present the analyses adjusted for maternal outcomes, highlighting significant differences between age groups. Compared to women aged 20–34 years, adolescents were less likely to receive guidance as to the reference maternity hospital (OR = 0.68), whereas women with AMA were more often guided in this regard (OR = 1.46). In addition, adolescents were more deprived of a companion during their stay in the maternity hospital (OR = 0.77).

**Table 4:** Odds ratio (OR), crude and adjusted^a^ for maternal outcomes according to women's age group. Rio de Janeiro, Brazil.

Maternal outcomes	Age group (years)					
	≤ 19			≥ 35 or over		
	Crude OR	Adjusted OR	95%CI	Crude OR	Adjusted OR	95%CI
Traveling	2.36	1.57	0.69–3.59	0.87	0.69	0.48–1.59
Guided on visiting the maternity hospital	**0.70**	**0.68**	**0.48–0.96**	**1.63**	**1.46**	**1.06–2.01**
Had a companion sometimes	**0.79**	**0.77**	**0.60–0.97**	0.69	0.96	0.67–1.35
Did not have a companion at all	0.98	1.37	0.57–3.27	1.17	1.35	0.65–2.83
Gestational hypertension	0.80	0.65	0.37–1.14	**1.47**	**1.78**	**1.14–2.79**
Gestational/pregestational diabetes	**0.26**	**0.26**	**0.12–0.53**	**2.09**	**1.87**	**1.21–3.61**
Placental abruption	4.22	3.10	0.53–18.14	**2.91**	**3.30**	**1.19–9.20**
Placenta accreta/increta/percreta	-	-	-	7.18	7.14	0.80–63.41
Diagnosis of maternal syphilis	1.04	0.98	0.61–1.57	**0.23**	**0.28**	**0.14–0.58**
Diagnosis of HIV	0.57	0.53	0.08–3.71	0.71	0.88	0.23–3.35
Severe maternal morbidity	1.05	0.88	0.50–1.55	**1.51**	**1.84**	**1.19–2.84**

95%CI: 95% confidence interval.

a The models of maternal outcomes were adjusted for the variables: skin color, level of education, parity, and residence region.

Reference group: 20-34 years and category "no" for maternal outcomes.

As for morbidities, adolescents showed 74% protection against gestational diabetes, whereas women with AMA had 72% protection against syphilis. Conversely, older women presented more chances of gestational hypertension (OR = 1.78), diabetes (OR = 1.87), placental abruption (OR = 3.30), and severe maternal morbidity (OR = 1.84).

In the perinatal outcomes (Table 5), despite the low frequency of perinatal death, it was more prevalent among newborns of adolescents (OR = 4.52) as well as spontaneous early-term delivery (OR = 1.47). In turn, adolescents showed a lower chance of early-term birth by cesarean section or induction (OR = 0.48).

**Table 5:** Odds ratio (OR), crude and adjusted^a^ for fetal and neonatal outcomes according to women's age group. Rio de Janeiro, Brazil.

Fetal and neonatal outcomes	Age group (years)					
	≤ 19			≥ 35 or over		
	Crude OR	Adjusted OR	95%CI	Crude OR	Adjusted OR	95%CI
Perinatal death	**4.04**	**4.52**	**1.13–18.08**	1.67	1.74	0.51–5.87
Newborn transferred to ICU/NICU	0.89	0.92	0.27–3.14	1.60	1.59	0.98–2.60
Congenital syphilis	1.07	0.90	0.37–2.22	**0.25**	**0.34**	**0.21–0.57**
Intrauterine growth restriction	2.43	2.06	0.54–7.89	1.35	1.66	0.57–4.83
5-minute Apgar index ≤ 7	1.48	1.33	0.75–2.38	**2.71**	**3.05**	**1.20–7.78**
Skin-to-skin contact in the delivery room	1.07	1.11	0.79–1.56	0.94	0.87	0.70–1.09
Breastfeeding in the first hour of life	1.13	0.97	0.68–1.40	0.75	0.83	0.53–1.29
Newborn was kept with the mother from birth to their room	1.41	1.20	0.79–1.83	0.70	0.89	0.61–1.29
Low birth weight	1.28	0.98	0.59–1.65	0.96	1.18	0.70–2.00
Spontaneous preterm birth	1.62	1.68	0.47–6.01	1.14	1.11	0.33–3.69
Induced preterm birth	1.34	1.60	0.54–4.74	1.88	1.52	0.70–3.29
Spontaneous early-term birth	**1.55**	**1.47**	**1.01–2.15**	1.37	1.52	0.82–2.80
Induced early-term birth	**0.27**	**0.48**	**0.23–0.98**	2.22	1.42	0.86–2.32

95%CI: 95% confidence interval.

a The fetal and neonatal outcome models were adjusted for the variables: skin color, level of education, parity, and residence region; and financing of delivery, in addition to those previously mentioned.

Reference group: 20-34 years and category "no" for fetal and neonatal outcomes.

Newborns of women with AMA showed a higher probability of 5-minute Apgar index < 7 (OR = 3.05). However, there was 66% protection against congenital syphilis in this group.

## DISCUSSION

Our results are consistent with the literature, demonstrating greater gestational and perinatal losses for adolescents and women with advanced maternal age when compared to those aged between 20 and 34 years. Among the main disadvantages observed, it is noteworthy that adolescents were less guided on the reference maternity hospital and more often deprived of a companion at all times in the maternity hospital. Women with AMA, in turn, had a higher prevalence of gestational hypertension, diabetes, placental abruption, and severe maternal morbidity. In addition, children of adolescents showed a higher probability of perinatal death and spontaneous early-term delivery, although they were protected against the early-term delivery by induction or antepartum cesarean section.

AMA newborns had a higher chance of presenting some degree of neonatal asphyxia, as indicated by the 5-minute Apgar index < 7. Preterm birth was estimated at 10.3%, with a higher concentration between the 34th and the 36th weeks of pregnancy. Early-term births accounted for almost one third of the total deliveries performed in the state of RJ.

The proportion of adolescent deliveries found in NBII in the state of RJ indicates a significant decrease in the proportion of births in women under 20 years of age in just over a decade, compared to that found at the national level in the Birth in Brazil survey (*Pesquisa Nascer no Brasil* – NB1), conducted between 2011 and 2012^15^, whose authors identified 18.6% of deliveries in adolescence^16^. This decline seems to reflect the impact of implemented public policies, which are not restricted to the biological aspects of pregnancy, but also aim to reduce socioeconomic losses, such as school dropout and early insertion in the labor market, usually poorly qualified. Examples of initiatives that may have contributed to this reduction include increasing access to contraceptive methods in primary health care and the School Health Program (*Programa Saúde na Escola*, an intersectoral initiative of the Brazilian Ministries of Health and Education aimed at integrating health and education actions for students of the public education system, promoting, preventing, and providing care for these students)^17,18^. As a result, there is also a decrease in negative perinatal outcomes among this population^5,6^.

In turn, NB1 identified 10.5% of women aged 35 years or over^16^, a considerably lower value than that found in the present study, which confirms a movement of pregnancy postponement in the state of RJ, following the increasing trend already observed in developed countries. This demographic acceleration, however, has generated concerns both in the clinical and public health fields, considering that AMA has been consistently associated with adverse pregnancy outcomes^7,19,20^.

We verified inequalities in care during prenatal care and delivery, highlighting that adolescents received less guidance as to visiting the reference maternity hospital, which hinders their link to the health facility. In contrast, this guidance was 63% more frequent in women with AMA compared to the age group of 20 to 34 years. The link of the pregnant woman to the maternity hospital where delivery will take place is a legal requirement in Brazil and one of the guidelines of the *Rede Cegonha* network^13^. This strategy is essential to avoid traveling in search of obstetric care, contributing to the reduction of cases of maternal near miss and neonatal deaths^21^.

The right to choose a full-time companion during hospitalization for delivery has been guaranteed by law since 2005^13^, contributing to the strengthening of the confidence and safety of women and reducing the experience of obstetric violence^22^. In 2023, this legislation was updated, expanding the rights of pregnant women^23^. In the NB1 Survey, only 18.7% of women had access to this right, with an even more unfavorable situation among adolescents, a population already more vulnerable^24^. According to these data, there was a significant improvement in access to a companion in the NBII-ERJ, compared to the last decade.

The chance of maternal-fetal complications increased in women with AMA — such as gestational hypertension and diabetes. The literature is unanimous in pointing to an increase in the prevalence of chronic diseases during pregnancy as maternal age progresses. Authors of a literature review, who addressed the implications for mother and newborn in pregnancies of women with AMA, found a higher risk of diabetes and hypertension, both chronic and gestational, from 35 years onwards, among other associated conditions^7^. Similarly, severe maternal morbidity was 50% more prevalent in women with AMA, a finding that corroborates previous studies such as Aoyama et al.^2^, carried out in Canada.

Advanced maternal age also increased the chance of placental abruption, in accordance with the review by Martinelli et al.^25^. Although the etiology remains uncertain, one of the theories suggests that atherosclerotic alterations occur in the blood vessels of the uterus, compromising the uteroplacental blood flow and resulting in large infarctions that cause hemorrhagic disorders in older women. Conversely, AMA women presented lower exposure to the diagnosis of syphilis during pregnancy and delivery, and their newborns presented lower proportions of congenital syphilis. These findings probably reflect the greater purchasing power of this group, evidenced by the greater presence of private financing of delivery (44.2% in AMA women *versus* 3.8% in adolescents), and by the high level of education, with one third of them having a higher education degree (additional data on level of education were not presented given the impossibility of comparison with adolescents).

Regarding fetal and neonatal outcomes, preterm birth did not remain significant in multiple analysis, contrary to findings of the NB1^26^, which may indicate improvement in prenatal care. However, adolescents presented a higher rate of perinatal deaths, in addition to a higher prevalence of spontaneous early-term births. In contrast, the probability of induced early-term delivery was significantly lower in this age group, which is in line with the results found in the NB1 study.

Despite the high prevalence and higher risk of complications, the consequences of early-term birth are still incipient, even though it represents almost one third of the deliveries in this study. According to scientific evidence, delivery between 37 and 38 weeks of gestation is associated with adverse outcomes for child health, such as greater need for hospitalization in NICU and greater occurrence of respiratory complications at birth, when compared to full-term births between 39 and 41 gestational weeks^9,27^. Supporting this evidence, the American College of Obstetricians and Gynecologists has recommended, since 2009, that elective cesarean sections should not be performed before 39 weeks of gestation, aiming to minimize neonatal complications associated with preterm birth^28^, a guidance also followed by the Brazilian Federal Council of Medicine^29^.

Among women with AMA, only the 5-minute Apgar index < 7 was more frequent than in the comparison group, as observed in the study by Glick et al.^9^. Furthermore, the transfer of newborns to the NICU was more common in this group. Although there was a borderline significance, authors of a cohort study conducted in France identified twice the rate of transfers of newborns to intensive neonatal care among women aged 40 years or older^30^.

These findings emphasize the challenges faced by adolescents in pregnancy and childbirth care and the major health complications in older women, stressing the need for special health policies to meet the specificities of each group. They also provide valuable information for decision-makers in public health, highlighting the urgency to expand strategies for preventing unplanned pregnancy, especially among adolescents, as well as a comprehensive guidance as to the possible risks associated with late pregnancies. Likewise, measures are essential to reduce obstetric traveling, promoting the link of pregnant women to the reference maternity hospitals, as recommended.

The described scenario highlights the urgency in the implementation of best practice guidelines for obstetric interventions, as well as public health policies aimed at reducing early-term births, especially those induced by antepartum cesarean sections, a critical problem to be faced in Brazil. Although the Brazilian Federal Council of Medicine recommends that elective cesarean sections not to be performed before 39 weeks of gestation^29^, we observed that less than 60% of cesarean sections occurred at full-term, not reaching half of deliveries in women aged 35 years or older.

As limitations, we mention the fact that the results do not apply to hospitals with less than one hundred deliveries per year. In addition, the variables related to complications derive from medical records, which depends on the quality of the records in each hospital, and the lack of records is considered as absence of complication. The main strength of the study lies in its design, which allowed the representation of the state of RJ both in the metropolitan region and in small cities, besides having a representative sample of postpartum women.

## Data Availability

The datasets generated and/or analyzed during the study are available from the corresponding author upon request.
