# Supplementary material for: Maternal and perinatal outcomes in adolescents and women with advanced maternal age
Source: Rev Saude Publica. 2025 Oct 20;59(Suppl 1):e9s. doi: 10.11606/s1518-8787.2025059006538 (PMC12539636; doi:10.11606/s1518-8787.2025059006538)
Supplement: Translation

# Translation: Desfechos maternos e perinatais em adolescentes e mulheres com idade materna avançada

**Authors:** Silvana Granado Nogueira da Gama, Arthur Orlando Corrêa Schilithz, Maria do Carmo Leal, Talita Teresa do Carmo, Mariza Miranda Theme-Filha, Denise Cavalcante de Barros, Glauce Cristine Ferreira Soares, Katrini Guidolini Martinelli

**Affiliations:** I Fundação Oswaldo Cruz. Escola Nacional de Saúde Pública. Rio de Janeiro, RJ, Brasil; II Instituto Nacional de Propriedade Industrial. Rio de Janeiro, RJ, Brasil; III Universidade de São Paulo. Escola de Artes, Ciências e Humanidades. São Paulo, SP, Brasil; IV Universidade Federal do Sul da Bahia. Centro de Formação em Ciências da Saúde. Teixeira de Freitas, BA, Brasil

**Keywords:** Idade Materna, Gravidez na Adolescência, Complicações na Gravidez, Resultado da Gravidez, Recé-Nascido Prematuro

## Abstract

**OBJETIVO:**

Analisar a associação entre extremos etários e desfechos maternos e perinatais em nascimentos ocorridos no estado do Rio de Janeiro, Brasil.

**MÉTODOS:**

Este estudo utilizou dados do projeto Nascer no Brasil II: Pesquisa Nacional Sobre Aborto, Parto e Nascimento (2021–2023). Foram incluídas 1.734 puérperas com recém-nascidos vivos ou natimortos, entrevistadas no pós-parto imediato. Informações foram obtidas por meio de entrevistas, prontuários clínicos e cartões de pré-natal. A análise empregou regressão logística múltipla para avaliar associações entre idade materna e desfechos, tendo como referência mulheres de 20–34 anos.

**RESULTADOS:**

Parto em adolescentes representou 10,1% das participantes, enquanto mulheres com idade materna avançada (IMA ≥ 35 anos) corresponderam a 18,0%. As adolescentes tiveram menor probabilidade de receber orientação para maternidade de referência (*odds ratio* — OR = 0,68) e de contar com acompanhante constante (OR = 0,77) e apresentaram menor prevalência de diabetes gestacional (OR = 0,26). Já mulheres com idade materna avançada foram mais frequentemente orientadas para maternidades de referência (OR = 1,46) e apresentaram maior risco de síndromes hipertensivas (OR = 1,78), diabetes gestacional (OR = 1,87), descolamento prematuro de placenta (OR = 3,30) e morbidade materna grave (OR = 1,84). Nos desfechos perinatais, adolescentes tiveram maior risco de óbito perinatal (OR = 4,52) e parto espontâneo a termo precoce (OR = 1,47), contudo menor probabilidade de termo precoce por intervenção obstétrica (OR = 0,48). Recém-nascidos de mulheres com idade materna avançada apresentaram maior risco de Apgar < 7 no quinto minuto (OR = 3,05) e menor chance de sífilis congênita (OR = 0,34).

**CONCLUSÃO:**

Adolescentes receberam pior assistência em relação às adultas, enquanto as mulheres com idade materna avançada apresentam maior frequência de complicações relacionadas à idade. Esses resultados destacam a necessidade de atenção diferenciada, especialmente diante do aumento de partos em mulheres com idade avançada.

## INTRODUÇÃO

As faixas etárias extremas da vida reprodutiva têm sido amplamente estudadas em função do aumento de risco de complicações obstétricas e perinatais. No Brasil, em 2022, cerca de 30% dos nascidos vivos (NV) foram de mulheres em extremos etários: 18,0% tinham idade materna avançada (IMA ≥ 35 anos) e 12,3% eram adolescentes (< 20 anos), conforme dados do Sistema de Informações sobre Nascidos Vivos (SINASC)^1^.

A postergação da maternidade tornou-se uma escolha comum entre mulheres nas últimas décadas, impulsionada pela busca de estabilidade financeira e avanço na carreira^2^. Contudo, a gravidez em mulheres com IMA está associada a maior risco de desfechos adversos, como hipertensão induzida pela gestação, hemorragia pré-parto, diabetes gestacional e pré-gestacional, além de anormalidades placentárias^3,4^. Nos desfechos perinatais, destacam-se maior incidência de natimortalidade, prematuridade, macrossomia e necessidade de internação em unidade de terapia intensiva neonatal (UTIN)^5,6^.

Apesar da redução na proporção de gravidez na adolescência, este grupo ainda demanda atenção diferenciada em razão dos maiores riscos de óbito perinatal, parto prematuro e restrição do crescimento fetal, especialmente nas adolescentes mais jovens^7–10^. Além disso, entre os desfechos maternos negativos se destacam anemia, doenças hipertensas da gestação, hemorragia pós-parto e pouco apoio socal^10,11^. O estudo também associa idades maternas extremas a maior risco de morbidade materna grave, incluindo condições potencialmente fatais, como o *near miss*, e de mortalidade materna^12^.

Ademais, desfechos como sífilis, HIV, morbidade materna grave, sífilis congênita, prematuridade e termo precoce segundo tipo de trabalho de parto ainda são pouco explorados, especialmente no contexto brasileiro. A política de humanização do parto implementada pela Rede Cegonha enfatiza tecnologias leves, como vinculação da gestante à maternidade, presença de acompanhante, contato pele a pele e amamentação precoce, para promover o bem-estar materno e neonatal^13^. Este estudo teve como objetivo analisar a associação entre extremos etários e desfechos maternos e perinatais em nascimentos no estado do Rio de Janeiro (RJ), Brasil, entre 2021 e 2023.

## MÉTODOS

Trata-se de uma análise do estudo "Nascer no Brasil II: Pesquisa Nacional Sobre Aborto, Parto e Nascimento (NBII)", que utilizou dados específicos do estado do RJ correspondentes ao período de 2021 a 2023.

O NBII tem como população de estudo mulheres internadas por motivo de parto ou aborto nos hospitais amostrados. A amostra foi selecionada em dois estágios. O primeiro estágio correspondia aos hospitais e levou em consideração o número de NV anuais, a macrorregião brasileira à qual eles pertenciam e a constatação do tipo de administração do hospital — público, privado ou misto. O segundo estágio da amostra dizia respeito às puérperas selecionadas para o estudo. Foram entrevistadas 30 e 50 puérperas nos hospitais com 100–499 e ≥ 500 NV/ano, respectivamente. Não foram incluídas na NB II aquelas com dificuldades de comunicação por distúrbio mental grave; que não compreendessem português; as surdas; as mulheres com gestações trigemelar ou mais; e as internadas para a realização de parto por determinação judicial. Pesos amostrais foram calculados com base nas probabilidades de inclusão e calibrados pelo SINASC, 2022. O protocolo do estudo NBII encontra-se publicado em Leal et al.^14^.

Para o presente estudo, que se concentra exclusivamente no estado do RJ, as puérperas foram distribuídas em 29 hospitais de 18 municípios. As entrevistas hospitalares ocorreram no pós-parto imediato, entre novembro de 2021 e junho de 2023, com as seguintes adaptações: ampliação da amostra (50 para 90 puérperas) nos hospitais públicos e mistos com ≥ 500 NV/ano e diferenciação entre o município do Rio de Janeiro (MRJ) e os demais municípios da região metropolitana (RM) e do interior (INT). A calibração foi feita utilizando os grupos compostos da combinação de estrato (que combina o quantitativo de partos anuais do estabelecimento, a macrorregião, o tipo de administração do hospital e a localização do hospital em região metropolitana ou interior), tipo de parto (vaginal, cesariana) e idade da mulher (< 20, 20–34, ≥ 35). Para essa análise, além dos critérios de elegibilidade já previstos para a NBII, também foram excluídas as mulheres internadas por aborto, as que se declararam indígenas e asiáticas, pelo pequeno número de casos, e as que tiveram gestações gemelares, por seu risco aumentado de desfechos adversos.

Foram utilizadas informações obtidas por meio da entrevista com a puérpera na internação, do cartão de pré-natal e do prontuário materno e neonatal. Os desfechos analisados foram coletados preferencialmente do cartão de pré-natal e do prontuário materno; quando não disponíveis, foi utilizada a entrevista com a gestante.

Esse estudo apresenta uma única variável de exposição: a idade materna (< 20 — adolescentes/ 20–34/ ≥ 35 anos — idade materna avançada). As demais variáveis independentes são apresentadas para a caracterização da população de estudo. Foram elas: cor da pele (branca/ preta ou parda); situação conjugal (sem companheiro/ com companheiro); índice de massa corporal (IMC) pré-gestacional (baixo peso/ eutrófico/ sobrepeso/ obesidade); nº partos anteriores (0/ 1/ ≥ 2); intenção de engravidar (tinha intenção de ficar grávida/ intenções variavam ou não eram muito claras/ não tinha intenção de ficar grávida); início do pré-natal (primeiro/ segundo ou terceiro trimestre/ não fez pré-natal); adequação do número de consultas de pré-natal para a idade gestacional (não/ sim); tipo de parto (vaginal/ cesariano); financiamento do parto (público/ misto/ privado); localização do hospital (região metropolitana/ interior).

Foram apresentados múltiplos desfechos, separados entre maternos e fetais. As variáveis dependentes que representam os desfechos maternos foram: peregrinação para a maternidade (não/sim); orientação a visitar a maternidade — vinculação (não/sim); presença de acompanhante (em todos os momentos/ em alguns momentos/ em nenhum momento); síndrome hipertensiva — hipertensão crônica, pré-eclâmpsia, eclâmpsia ou síndrome HELLP (não/sim); diabetes gestacional ou pré-gestacional (não/sim); descolamento prematuro de placenta (não/sim); placenta acreta/increta/percreta (não/sim); diagnóstico de sífilis materna (não/sim); infecção por HIV (não/sim) e morbidade materna grave — condições potencialmente ameaçadoras da vida ou *near miss* materno (não/sim).

Os desfechos fetais e neonatais inseridos na análise foram: óbito perinatal (não/ sim); sífilis congênita (não/sim); crescimento intrauterino restrito (não/sim); baixo peso ao nascer < 2.500 gramas (não/sim); idade gestacional no nascimento (≤ 33 semanas/ 34–36 semanas/ 37–38 semanas/ ≥ 39 semanas); tipo de termo no nascimento (≥ 39 semanas/ prematuro espontâneo/ prematuro induzido ou cesariana anteparto/ termo precoce espontâneo/ termo precoce induzido ou cesariana anteparto); Apgar no quinto minuto < 7 (não /sim); contato pele a pele na sala de parto (não/sim); amamentação na primeira hora (não/sim); recém-nascido com a mãe desde o nascimento até o alojamento conjunto ou quarto (não/sim); internação na UTIN ou transferência do recém-nascido para ela (não/sim).

Foram apresentadas as frequências absolutas e relativas de características maternas, desfechos maternos, fetais e neonatais. As diferenças de proporções entre os grupos (faixas etárias maternas) foram testadas por meio do χ^2^ com ajuste de Rao-Scott e intervalo de confiança de 95% (IC95%).

Foram elaborados modelos de regressão logísticos uni e multivariados para analisar a associação entre a idade materna (exposição) e desfechos maternos, fetais e neonatais por meio de razão de chances (OR) bruta e ajustada. Para controle de potencial confundimento, as variáveis de ajuste foram selecionadas por meio da plausibilidade teórica. O ajuste do modelo multivariado para os desfechos maternos foi composto das variáveis: cor da pele, número de partos anteriores e região de moradia. Para os desfechos fetais e neonatais, o ajuste foi feito por: cor da pele, número de partos anteriores, região de moradia e financiamento do parto.

O desenho complexo da amostragem foi levado em consideração durante toda a análise estatística. Cada estrato de seleção recebeu um procedimento de calibração por razão de pesos amostrais básicos para assegurar que a distribuição das puérperas fosse semelhante à observada nos nascimentos da população amostrada em 2022, derivando percentuais ponderados.

## RESULTADOS

Foram incluídas nas análises 1.734 puérperas no pós-parto imediato, sendo 10,1% adolescentes e 18,0% com idade materna avançada. A maioria das entrevistadas se declarou preta/parda (69,6%), com companheiro (81,1%), eutrófica (41,9%), sem parto anterior (43,7%), tendo iniciado o pré-natal no primeiro trimestre (78,6%), com número de consultas pré-natal adequado para a idade gestacional (67,3%), com financiamento público do parto (63,3%), parto em hospitais da região metropolitana (76,1%) e cesariana como tipo de parto (58,5%) (Tabela 1).

**Tabela 1:** Características sociodemográficas e reprodutivas de puérperas segundo faixa etária materna. Rio de Janeiro, Brasil.

Características		Faixa etária (anos)			Total (n = 1.732)	Valor p^a^
		< 20 (n = 176)	20–34 (n = 1.228)	≥ 35 (n = 312)		
		%	%	%	%	
**Adequação de consultas pré-natal (n = 1.656)**						
	não	76 (44,2)	401(33,7)	65(22,0)	542(32,7)	0,002
	sim	96(55,8)	788(66,3)	230(78,0)	1.114 (67,3)	
**Tipo de parto (n = 1.733)**						
	vaginal	107 (58,8)	526 (42,5)	86 (27,6)	719 (41,5)	< 0,0001
	cesariana	75 (41,2)	712 (57,5)	226 (72,4)	1.013 (58,5)	
**Financiamento do parto (n = 1.734)**						
	público	145 (79,7)	812 (65,5)	140 (44,9)	1.097 (63,3)	< 0,0001
	misto	30 (16,5)	154 (12,4)	34 (10,9)	218 (12,6)	
	privado	7 (3,8)	273 (22,1)	138 (44,2)	418 (24,1)	
**Localização do hospital (n = 1.922)**						
	região metropolitana	138 (75,8)	935 (75,5)	246 (78,6)	1.319 (76,1)	0,561
	interior	44 (24,2)	304 (24,5)	67 (21,4)	415 (23,9)	

IMC: índice de massa corporal (kg/m^2^).

a Valor p do teste χ^2^ com ajuste de Rao-Scott.

Ao comparar os extremos etários, as adolescentes apresentaram percentuais maiores e significativos para cor da pele preta/parda, sem companheiro, baixo peso, não intenção de engravidar, sem parto anterior, início do pré-natal no segundo/ terceiro trimestre, financiamento público do parto e parto vaginal. Já as IMA eram majoritariamente de pele branca, com companheiro, sobrepeso ou obesas, tinham mais intenção de engravidar, dois ou mais partos anteriores, iniciaram o pré-natal no primeiro trimestre, tiveram maior adequação no número de consultas, com financiamento do parto privado e mais cesarianas.

Na Tabela 2 são expostos as morbidades gestacionais e os desfechos maternos pela faixa etária. A peregrinação em busca de atendimento para o parto foi identificada em 12,1% das entrevistadas, valor que quase dobra entre as adolescentes, com 23,6%. Apenas 17,7% das puérperas referiram ter sido encaminhadas a visitar a maternidade de referência para o parto, resultado pior entre as adolescentes, de 12,6%. A presença de acompanhante em todos os momentos da internação da mulher foi relatada por 70,5% das puérperas, valor semelhante entre os grupos etários. A síndrome hipertensiva foi identificada em 16,1% das entrevistadas, sendo menor nas mais jovens (12,6%) e mais elevada entre as de mais idade (21,1%). Do mesmo modo, a diabetes foi mais frequente nas IMA (13,7%), assim como a morbidade materna grave (18,2%). A ocorrência de placenta acreta/ increta/ percreta foi baixa, identificada em 0,5% das mulheres. O diagnóstico de sífilis materna foi de 14,3%, com maior frequência nas adolescentes, 17,0%

**Tabela 2:** Morbidades gestacionais e desfechos maternos segundo faixa etária da puérpera. Rio de Janeiro, Brasil.

Desfechos maternos		Faixa etária			Total (n = 1.732)	Valor p^a^
		< 20 (n = 176)	20–34 (n = 1.228)	≥ 35 (n = 312)		
		%	%	%	%	
**Descolamento prematuro de placenta (n = 1.732)**						
	não	176 (97,2)	1.230 (99,3)	306 (98,1)	1.712 (98,8)	0,080
	sim	5 (2,8)	9 (0,7)	6 (1,9)	20 (1,2)	
**Placenta acreta/increta/percreta (n = 1.734)**						
	não	182 (100,0)	1.236 (99,8)	307 (98,1)	1.725 (99,5)	0,003
	sim	0 (0,0)	3 (0,2)	6 (1,9)	9 (0,5)	
**Diagnóstico de sífilis materna (n = 1.733)**						
	não	151 (83,0)	1.036 (83,6)	299 (95,8)	1.486 (85,7)	< 0,001
	sim	31 (17,0)	203 (16,4)	13 (4,2)	247 (14,3)	
**Infecção por HIV (n = 1.734)**						
	não	181 (99,5)	1.228 (99,1)	311 (99,4)	1.720 (99,2)	0,687
	sim	1 (0,5)	11 (0,9)	2 (0,6)	14 (0,8)	
**Morbidade materna grave (1.733)**						
	não	157 (86,7)	1.080 (87,2)	256 (81,8)	1.493 (86,2)	0,107
	sim	24 (13,3)	159 (12,8)	57 (18,2)	240 (13,8)	

Síndromes hipertensivas: hipertensão crônica, DHEG, pré-eclâmpsia, eclâmpsia e HELLP.

Morbidade materna grave: condições potencialmente ameaçadoras da vida ou near miss materno

a Valor p do teste χ^2^ com ajuste de Rao-Scott.

Com relação aos desfechos perinatais, observou-se que 1,2% dos nascimentos resultou em óbito perinatal, sendo essa percentagem mais alta entre as adolescentes (3,3%), embora sem significância estatística. A sífilis congênita foi diagnosticada em 7,9% dos casos, com prevalência maior entre os nascimentos de adolescentes (9,4%). A proporção de prematuridade (< 37ª semana gestacional) foi de 10,3%. Contudo, a prematuridade antes da 33ª semana foi mais comum entre as adolescentes (6,1%), enquanto nas IMA foi mais frequente o nascimento pré-termo tardio (42,6%). Entre as adolescentes, a prematuridade espontânea foi mais prevalente (7,2%), enquanto nas IMA prevaleceu a prematuridade por indução ou cesariana anteparto (7,3%).

A prevalência do índice de Apgar < 7 no quinto minuto foi semelhante entre os grupos etários. O contato pele a pele na sala de parto e a amamentação no primeiro minuto ocorreram em menos de 60% dos casos, sem diferirem estatisticamente entre os grupos etários. Quanto à permanência dos recém-nascidos com as mães desde o nascimento até a chegada ao alojamento, 65,5% das entrevistadas relataram ter ficado com seus filhos, com maior proporção observada entre as adolescentes (73,6%). De maneira semelhante, a internação em UTIN foi menos frequente entre os filhos de adolescentes e mais prevalente entre os filhos de mulheres com 35 anos ou mais, porém sem significância estatística (Tabela 3).

**Tabela 3:** Desfechos fetais e neonatais segundo faixa etária da puérpera. Rio de Janeiro, Brasil.

Desfechos fetais e neonatais		Faixa etária			Total (n = 1.732)	Valor p^a^
		< 20 (n = 182)	20–34 (n = 1.228)	≥ 35 (n = 312)		
		%	%	%	%	
**Óbito Perinatal (n = 1.732)**						
	Sim	6 (3,3)	10 (0,8)	4 (1,3)	20 (1,2)	0,095
	Não	176 (96,7)	1.228 (99,2)	308 (98,7)	1.712 (98,8)	
**Sífilis congênita (n = 1.732)**						
	sim	17 (9,4)	111 (9,0)	8 (2,6)	136 (7,9)	0,031
	não	164 (90,6)	1.127 (91,0)	305 (97,4)	1.596 (92,1)	
**Crescimento Intrauterino Restrito (n = 1.732)**						
	sim	4 (2,2)	12 (1,0)	4 (1,3)	20 (1,2)	0,285
	não	177 (97,8)	1.227 (99,0)	308 (98,7)	1.712 (98,8)	
**Baixo Peso ao Nascer (< 2.500 gramas) (n = 1.733)**						
	sim	21 (11,5)	113 (9,1)	28 (8,9)	162 (9,3)	0,596
	não	161 (88,5)	1.125 (90,9)	285 (91,1)	1.571 (90,7)	
**Semanas gestacionais (n = 1.734) (semanas)**						
	≤ 33	11 (6,1)	20 (1,6)	10 (3,2)	41 (2,4)	
	34–36	14 (7,7)	98 (7,9)	25 (8,0)	137 (7,9)	0,022
	37–38	47 (26,0)	375 (30,3)	133 (42,6)	555 (32,0)	
	≥ 39	109 (60,2)	746 (60,2)	144 (46,2)	999 (57,7)	
**Tipo de prematuridade (n=1.732)**						
	≥39 semanas	109 (60,6)	746 (60,2)	144 (46,0)	999 (57,7)	
	Prematuro espontâneo	13 (7,2)	56 (4,5)	12 (3,8)	81 (4,7)	
	Prematuro induzido ou cesariana anteparto	12 (6,7)	62 (5,0)	23 (7,3)	97 (5,6)	0,015
	Termo precoce espontâneo	38 (21,1)	168 (13,6)	45 (14,4)	251 (14,5)	
	Termo precoce induzido ou cesariana anteparto	8 (4,4)	207 (16,7)	89 (28,4)	304 (17,6)	
**Índice de Apgar no quinto minuto (n = 1.722)** b						
	≥ 7	170 (96,6)	1.208 (97,7)	292 (94,2)	1.670 (97,0)	0,125
	< 7	6 (3,4)	28 (2,3)	18 (5,8)	52 (3,0)	
**Contato pele a pele na sala de parto (n = 1.718)** b						
	sim	105 (59,7)	716 (58,1)	176 (56,8)	997 (58,0)	0,752
	não	71 (40,3)	516 (41,9)	134 (43,2)	721 (42,0)	
**Amamentação nas primeiras 24 h (n = 1.714)** b						
	sim	108 (61,0)	714 (58,1)	157 (51,0)	979 (57,1)	0,265
	não	69 (39,0)	515 (41,9)	151 (49,0)	735 (42,9)	
**RN com a mãe do nascimento até a chegada ao alojamento (n = 1.712)** b						
	sim	128 (73,6)	813 (66,3)	181 (58,0)	1.122 (65,5)	0,005
	não	46 (26,4)	413 (33,7)	131 (42,0)	590 (34,5)	
**Internação em UTI/transferência do RN (n = 1.724)** b						
	sim	13 (7,3)	99 (8,0)	38 (12,2)	150 (8,7)	0,286
	não	164 (92,7)	1.137 (92,0)	273 (87,8)	1.574 (91,3)	

Síndromes hipertensivas: hipertensão crônica, DHEG, pré-eclâmpsia, eclâmpsia e HELLP.

Morbidade materna grave: condições potencialmente ameaçadoras da vida ou *near miss* materno.

a Valor p do teste χ^2^ com ajuste de Rao-Scott.

b Apenas para mulheres com nascidos vivos.

Na Tabela 4 são apresentadas as análises ajustadas para desfechos maternos, destacando-se diferenças significativas entre os grupos etários. Em comparação com mulheres de 20–34 anos, adolescentes tiveram menor probabilidade de receber orientação para a maternidade de referência (OR = 0,68), enquanto mulheres de IMA foram mais frequentemente orientadas (OR = 1,46). Além disso, adolescentes foram mais privadas de acompanhante durante toda a permanência na maternidade (OR = 0,77).

**Tabela 4:** *Odds ratio* (OR) brutos e ajustados^a^ para os desfechos maternos segundo a faixa etária da mulher. Rio de Janeiro, Brasil.

Desfechos maternos	Faixa etária (anos)					
	≤ 19			≥ 35 ou mais		
	OR bruta	OR ajustada	IC 95%	OR bruta	OR ajustada	IC 95%
Peregrinação	2,36	1,57	0,69–3,59	0,87	0,69	0,48–1,59
Orientada a visitar a maternidade	**0,70**	**0,68**	**0,48–0,96**	**1,63**	**1,46**	**1,06–2,01**
Acompanhante em alguns momentos	**0,79**	**0,77**	**0,60–0,97**	0,69	0,96	0,67–1,35
Acompanhante em nenhum momento	0,98	1,37	0,57–3,27	1,17	1,35	0,65–2,83
Síndromes hipertensivas	0,80	0,65	0,37–1,14	**1,47**	**1,78**	**1,14–2,79**
Diabetes gestacional/pré-gestacional	**0,26**	**0,26**	**0,12–0,53**	**2,09**	**1,87**	**1,21–3,61**
Descolamento prematuro de placenta	4,22	3,10	0,53–18,14	**2,91**	**3,30**	**1,19–9,20**
Placenta acreta/increta/percreta	-	-	-	7,18	7,14	0,80–63,41
Diagnóstico de sífilis materna	1,04	0,98	0,61–1,57	**0,23**	**0,28**	**0,14–0,58**
Diagnóstico de HIV	0,57	0,53	0,08–3,71	0,71	0,88	0,23–3,35
Morbidade materna grave	1,05	0,88	0,50–1,55	**1,51**	**1,84**	**1,19–2,84**

IC95%: intervalo de 95% de confiança.

a Os modelos dos desfechos maternos foram ajustados pelas variáveis: cor da pele, escolaridade, paridade e região de moradia.

Grupo de referência: 20–34 anos e categoria não para os desfechos maternos.

Quanto às morbidades, adolescentes mostraram proteção de 74% contra diabetes gestacional, enquanto mulheres com IMA tiveram proteção de 72% contra sífilis. Por outro lado, as mulheres de maior idade apresentaram mais chances de síndromes hipertensivas (OR = 1,78), diabetes (OR = 1,87), descolamento prematuro de placenta (OR = 3,30) e morbidade materna grave (OR = 1,84).

Nos desfechos perinatais (Tabela 5), apesar da baixa frequência de óbito perinatal, este foi mais prevalente entre recém-nascidos de adolescentes (OR = 4,52), assim como o parto a termo precoce espontâneo (OR = 1,47). Por outro lado, adolescentes mostraram menor chance de nascimento a termo precoce por cesariana ou indução (OR = 0,48).

**Tabela 5:** *Odds ratio* (OR) brutos e ajustados^a^ para os desfechos fetais e neonatais segundo a faixa etária da mulher. Rio de Janeiro, Brasil.

Desfechos fetais e neonatais	Faixa etária (anos)					
	≤ 19			≥ 35 ou mais		
	OR bruta	OR ajustada	IC95%	OR bruta	OR ajustada	IC95%
Óbito perinatal	**4,04**	**4,52**	**1,13–18,08**	1,67	1,74	0,51–5,87
RN transferido para UTI/UTIN	0,89	0,92	0,27–3,14	1,60	1,59	0,98–2,60
Sífilis congênita	1,07	0,90	0,37–2,22	**0,25**	**0,34**	**0,21–0,57**
Crescimento intrauterino restrito (CIUR)	2,43	2,06	0,54–7,89	1,35	1,66	0,57–4,83
Índice de Apgar no quinto minuto ≤ 7	1,48	1,33	0,75–2,38	**2,71**	**3,05**	**1,20–7,78**
Contato pele a pele na sala de parto	1,07	1,11	0,79–1,56	0,94	0,87	0,70–1,09
Aleitamento na primeira hora de vida	1,13	0,97	0,68–1,40	0,75	0,83	0,53–1,29
RN c/ mãe do nascimento até alojamento	1,41	1,20	0,79–1,83	0,70	0,89	0,61–1,29
Baixo peso ao nascer	1,28	0,98	0,59–1,65	0,96	1,18	0,70–2,00
Prematuro espontâneo	1,62	1,68	0,47–6,01	1,14	1,11	0,33–3,69
Prematuro induzido	1,34	1,60	0,54–4,74	1,88	1,52	0,70–3,29
Termo precoce espontâneo	**1,55**	**1,47**	**1,01–2,15**	1,37	1,52	0,82–2,80
Termo precoce induzido	**0,27**	**0,48**	**0,23–0,98**	2,22	1,42	0,86–2,32

IC95%: intervalo de 95% de confiança.

a Os modelos fetais e neonatais dos desfechos foram ajustados pelas variáveis: cor da pele, escolaridade, paridade e região de moradia; e o financiamento do parto, além das citadas anteriormente.

Grupo de referência: 20–34 anos e categoria não para os desfechos fetais e neonatais.

Recém-nascidos de mulheres com IMA apresentaram maior probabilidade de índice de Apgar < 7 no quinto minuto (OR = 3,05). Contudo, houve proteção de 66% contra sífilis congênita neste grupo.

## DISCUSSÃO

Os resultados deste estudo são consistentes com a literatura, revelando maiores prejuízos gestacionais e perinatais para adolescentes e mulheres com idade materna avançada quando comparadas àquelas com idades entre 20 e 34 anos. Entre as principais desvantagens observadas, destaca-se que as adolescentes foram menos orientadas para a maternidade de referência e mais frequentemente privadas de acompanhante integral na maternidade. As mulheres com IMA, por sua vez, apresentaram maior prevalência de síndromes hipertensivas, diabetes, descolamento prematuro da placenta e morbidade materna grave. Além disso, filhos de adolescentes mostraram maior probabilidade de óbito perinatal e nascimento a termo precoce espontâneo, embora estivessem protegidas contra o termo precoce induzido ou por cesariana anteparto.

Os recém-nascidos de IMA tiveram maior chance de apresentar algum grau de asfixia neonatal, como indicado por um Apgar < 7 no quinto minuto. Estimou-se proporção de prematuridade de 10,3%, com maior concentração entre a 34ª e a 36ª semana de gestação. Os nascimentos a termo precoce representaram quase um terço do total de partos realizados no estado do RJ.

A proporção de partos na adolescência encontrada no NBII no estado do RJ aponta significativa queda na proporção de partos em mulheres com menos de 20 anos em pouco mais de uma década, comparada ao encontrado em nível nacional na Pesquisa Nascer no Brasil (NB1), realizada entre 2011 e 2012^15^, que identificou 18,6% de partos na adolescência^16^. Esse declínio parece refletir o impacto das políticas públicas implementadas, que não se restringem aos aspectos biológicos da gravidez, mas também visam reduzir os prejuízos socioeconômicos, como a evasão escolar e a inserção precoce no mercado de trabalho, geralmente pouco qualificado. Exemplos de iniciativas que podem ter contribuído para essa redução incluem a ampliação do acesso a métodos contraceptivos na atenção primária e o Programa Saúde na Escola^17,18^. Como resultado, também se observa diminuição nos desfechos perinatais negativos entre essa população^5,6^.

Por sua vez, o NB1 identificou 10,5% de mulheres com 35 anos ou mais^16^, valor consideravelmente inferior ao encontrado no presente estudo, o que confirma um movimento de postergação da gravidez no estado do RJ, seguindo a tendência crescente já observada nos países desenvolvidos. Essa aceleração demográfica, no entanto, tem gerado preocupações tanto na área clínica quanto na saúde pública, uma vez que a IMA tem sido consistentemente associada a resultados adversos na gestação^7,19,20^.

O estudo revelou desigualdades nos cuidados durante o pré-natal e o parto, destacando que as adolescentes receberam menos orientações sobre a visita à maternidade de referência, o que dificulta seu vínculo com a unidade de saúde. Em contraste, essa orientação foi 63% mais frequente nas mulheres com IMA em comparação ao grupo de 20 a 34 anos. A vinculação da gestante à maternidade onde ocorrerá o parto é uma exigência legal no Brasil e uma das diretrizes da Rede Cegonha^13^. Essa estratégia é fundamental para evitar a peregrinação na busca por atendimento obstétrico, contribuindo para a redução de casos de *near miss* materno e óbitos neonatais^21^.

O direito à escolha do acompanhante em tempo integral durante a internação para o parto é garantido por lei desde 2005^13^, contribuindo para o fortalecimento da confiança e segurança da mulher, além de reduzir a experiência de violência obstétrica^22^. Em 2023, essa legislação foi atualizada, ampliando os direitos das gestantes^23^. No estudo NB1, apenas 18,7% das mulheres tiveram acesso a esse direito, com situação ainda mais desfavorável entre as adolescentes, população já mais vulnerabilizada^24^. Esses dados demonstram melhoria expressiva no acesso ao acompanhante no NBII-ERJ, em comparação à última década.

A chance de complicações materno-fetais aumentou nas mulheres com IMA, como as síndromes hipertensivas e a diabetes. A literatura é unânime ao apontar o aumento da prevalência de doenças crônicas na gestação à medida que a idade materna avança. Um artigo de revisão que abordou as implicações para mãe e recém-nascido em gestações de mulheres com IMA encontrou maior risco de diabetes e síndromes hipertensivas, tanto crônicas quanto gestacionais, dos 35 anos em diante, entre outras condições associadas^7^. De maneira semelhante, a morbidade materna grave foi 50% mais prevalente nas mulheres com IMA, um achado que corrobora estudos anteriores, como o de Aoyama et al.^2^, realizado no Canadá.

A idade materna avançada também aumentou a chance de descolamento prematuro da placenta, em concordância com a revisão de Martinelli et al.^25^. Embora a etiologia permaneça incerta, uma das teorias sugere que ocorrem alterações ateroscleróticas nos vasos sanguíneos do útero, comprometendo o fluxo uteroplacentário e resultando em grandes infartos que causam distúrbios hemorrágicos em mulheres mais velhas. Por outro lado, as mulheres IMA apresentaram menor exposição ao diagnóstico de sífilis durante a gestação e parto, assim como seus conceptos apresentaram menores proporções de sífilis congênita. Esses achados provavelmente refletem o maior poder aquisitivo desse grupo, evidenciado pela maior presença de financiamento privado do parto (44,2% nas mulheres IMA *versus* 3,8% nas adolescentes), e pelo elevado grau de escolaridade, com um terço delas tendo nível superior completo (dados adicionais sobre escolaridade não apresentados dada a impossibilidade de comparação com as adolescentes).

Quanto aos desfechos fetais e neonatais, a prematuridade não se manteve significativa na análise múltipla, indo de encontro aos achados no NB1^26^, o que pode indicar melhoria no cuidado pré-natal. No entanto, as adolescentes apresentaram taxa mais elevada de óbitos perinatais, além de maior prevalência de nascimentos a termo precoce espontâneos. Em contraponto, a probabilidade de parto a termo precoce induzido foi significativamente menor nesse grupo etário, o que está alinhado com os resultados encontrados no estudo NB1.

Apesar da elevada prevalência e do maior risco de complicações, as consequências do nascimento a termo precoce ainda recebem pouca atenção, mesmo representando quase um terço dos partos neste estudo. Evidências científicas revelam que o parto entre 37 e 38 semanas de gestação está associado a desfechos adversos para a saúde infantil, como maior necessidade de internação em UTIN e maior ocorrência de complicações respiratórias ao nascer, quando comparado aos nascimentos a termo completo entre 39 e 41 semanas gestacionais^9,27^. Em apoio a essa evidência, o Colégio Americano de Obstetras e Ginecologistas recomenda, desde 2009, que cesarianas eletivas não sejam realizadas antes de 39 semanas de gestação, visando minimizar complicações neonatais associadas à prematuridade^28^, orientação também seguida pelo Conselho Federal de Medicina^29^.

Entre as mulheres com IMA, apenas o índice de Apgar < 7 no quinto minuto foi mais frequente que no grupo de comparação, tal como observado no estudo de Glick et al.^9^. Além disso, a transferência de recém-nascidos para a UTIN foi mais comum nesse grupo. Embora a significância tenha sido limítrofe, um estudo de coorte conduzido na França identificou taxa duas vezes maior de transferências de recém-nascidos para cuidados intensivos neonatais entre mulheres com 40 anos ou mais^30^.

Esses achados reforçam os desafios enfrentados por adolescentes na assistência à gestação e parto e as maiores complicações de saúde em mulheres de idade avançada, reforçando a necessidade de políticas de saúde diferenciadas para atender às especificidades de cada grupo. Ainda fornecem informações valiosas para os tomadores de decisão em saúde pública, destacando a premência em ampliar as estratégias para a prevenção da gravidez não planejada, especialmente entre as adolescentes, além de uma orientação abrangente sobre os possíveis riscos associados às gestações tardias. Igualmente, são essenciais medidas para reduzir a peregrinação obstétrica, promovendo a vinculação das gestantes às maternidades de referência, conforme preconizado.

O cenário descrito destaca a urgência na implementação de diretrizes de melhores práticas para intervenções obstétricas, assim como políticas de saúde pública voltadas para a redução de nascimentos a termo precoce, especialmente os induzidos por cesarianas anteparto, um problema crítico a ser enfrentado no Brasil. Embora o Conselho Federal de Medicina tenha recomendado a não realização de cesarianas eletivas antes das 39 semanas de gestação^29^, observou-se que menos de 60% das cesarianas ocorreram a termo completo, não alcançando a metade dos partos nas mulheres com 35 anos ou mais.

Como limitações, os resultados não se aplicam a hospitais com menos de cem partos por ano. Além disso, as variáveis relacionadas às complicações são provenientes de prontuários médicos, o que depende da qualidade dos registros em cada hospital, sendo o não registro considerado como ausência de complicação. A principal força do estudo reside em seu desenho, que possibilitou a representação do estado do RJ tanto na região metropolitana quanto no interior, além de contar com uma amostra representativa de puérperas.

## Data Availability

Os conjuntos de dados gerados e/ou analisados durante o estudo estão disponíveis com o autor correspondente mediante solicitação.
